# TMPRSS4, a type II transmembrane serine protease, as a potential therapeutic target in cancer

**DOI:** 10.1038/s12276-023-00975-5

**Published:** 2023-04-03

**Authors:** Semi Kim

**Affiliations:** 1 Microbiome Convergence Research Center, Korea Research Institute of Bioscience and Biotechnology, Daejon, 34141 Korea; 2 Department of Functional Genomics, Korea University of Science and Technology, Daejon, 34113 Korea

**Keywords:** Oncogenes, Tumour biomarkers, Metastasis

## Abstract

Proteases are involved in almost all biological processes, implying their importance for both health and pathological conditions. Dysregulation of proteases is a key event in cancer. Initially, research identified their role in invasion and metastasis, but more recent studies have shown that proteases are involved in all stages of cancer development and progression, both directly through proteolytic activity and indirectly via regulation of cellular signaling and functions. Over the past two decades, a novel subfamily of serine proteases called type II transmembrane serine proteases (TTSPs) has been identified. Many TTSPs are overexpressed by a variety of tumors and are potential novel markers of tumor development and progression; these TTSPs are possible molecular targets for anticancer therapeutics. The transmembrane protease serine 4 (TMPRSS4), a member of the TTSP family, is upregulated in pancreatic, colorectal, gastric, lung, thyroid, prostate, and several other cancers; indeed, elevated expression of TMPRSS4 often correlates with poor prognosis. Based on its broad expression profile in cancer, TMPRSS4 has been the focus of attention in anticancer research. This review summarizes up-to-date information regarding the expression, regulation, and clinical relevance of TMPRSS4, as well as its role in pathological contexts, particularly in cancer. It also provides a general overview of epithelial-mesenchymal transition and TTSPs.

## Cancer: Possible enzyme target for treatment

Targeting a protein-degrading enzyme involved in the growth of solid tumours and the progression of multiple cancers could prove a valuable therapeutic approach. Dysregulation of a ubiquitous family of enzymes called the transmembrane serine proteases is a common feature of cancers. These proteases are directly involved in tumour development and disease progression. Semi Kim at the Korea Research Institute of Bioscience and Biotechnology in Daejon, South Korea, reviewed the role of transmembrane protease serine 4 (TMPRSS4), which is over-expressed in multiple cancers and correlates with poor prognosis. Recent studies show that blocking TMPRSS4 activity reduced cell migration and invasion, and inhibited tumour growth in lung and prostate cancer. The mechanisms behind TMPRSS4 upregulation remain unclear, and further investigations are required into how its expression is modulated in health and disease.

## Epithelial-mesenchymal transition

Epithelial-mesenchymal transition (EMT) is a reversible cellular process during which organized epithelial cells lose apical-basal polarity, decrease cell-cell adhesion, and reorganize their cytoskeleton while simultaneously individually or collectively acquiring mesenchymal features such as fibroblast-like morphology, increased cell motility, and invasive capability^1–3^. Cells undergoing EMT show reduced expression or function of epithelial markers such as E-cadherin, ZO-1, and certain cytokeratins, along with increased expression of mesenchymal markers such as vimentin, fibronectin, N-cadherin, and certain integrins.

EMT plays critical roles during developmental processes, including gastrulation, somitogenesis, and neural crest migration, as well as during epithelial wound healing in adult organisms^1^. Furthermore, EMT is activated during pathological processes such as cancer and organ fibrosis.

In cancer cells, EMT is rarely activated through a cell-autonomous process. Rather, EMT is instigated by paracrine signaling factors derived from the tumor-associated reactive stroma; notably, EMT events are induced by transforming growth factor-β (TGF-β) and other factors, including WNT proteins, hypoxia, growth factors, cytokines, and extracellular matrix (ECM)-integrin adhesions^4,5^. Consequently, these factors induce the expression and functional activation of EMT-inducing transcription factors (EMT-TFs), which can pleiotropically orchestrate diverse transcriptional changes associated with EMT. The major group of EMT-TFs includes members of the zinc-finger-binding transcription factors (i.e., the SNAIL family: SNAIL encoded by SNAI1 and SLUG encoded by SNAI2), the TWIST family of basic helix-loop-helix factors (TWIST1 and TWIST2), and the zinc-finger E-box-binding homeobox factors (the ZEB family: ZEB1 and ZEB2). Other transcription factors, such as Forkhead box C (FOXC2) and paired-related homeobox 1 (PRRX1), can also induce EMT^6^. These factors display distinct expression profiles and target gene patterns depending on the cell and tissue type.

In addition, proteases are involved in EMT induction^5,7^; for example, matrix metalloproteinase (MMP)−3 triggers EMT by increasing the cellular levels of reactive oxygen species (ROS), leading to upregulation of SNAIL. MMP-14 and MMP-8 induce EMT through activation of TGF-β signaling. IL-6-induction of a disintegrin and metalloprotease (ADAM) 9 expression leads to ROS production and EMT. ADAM10 cleaves the ectodomain of E-cadherin, which modulates cell-cell adhesion, cell migration, β-catenin translocation/signaling, and EMT^7^. The membrane-bound serine protease TMPRSS4 induces EMT in colon and hepatocellular carcinoma cells, which is accompanied by upregulation of ZEB2, SNAIL, and SLUG^8,9^. TMPRSS3 induces EMT in gastric cancer cells through regulation of the ERK1/2 and AKT pathways^10^. TMPRSS2 induces proinvasive EMT and metastasis in a prostate cancer model, potentially through prohepatocyte growth factor (HGF) activation and subsequent c-MET signaling^11^.

EMT-TFs are additionally regulated by miRNAs (miRs) and long noncoding RNAs (lncRNAs), epigenetic modifications, alternative splicing, and posttranslational regulation of protein stability. For example, miR-200s and ZEB1/2 form a double-negative feedback loop in which ZEB1/2 inhibits the transcription of miR-200 mRNAs, and miR-200s suppress ZEB1 expression. Similarly, a double-negative feedback loop is established between miR-1 and miR-200 and SNAI2. Such mechanisms allow rapid and robust enforcement of epithelial and mesenchymal states in response to minimal signals^1^. In addition, ZEB1 can switch from a transcriptional repressor to an activator by interacting with YAP1^12^ or the coactivators PCAF and p300^1^. ZEB2 cooperates with SP1 to function as a transcriptional activator to promote expression of the mesenchymal markers integrin α5 and vimentin^13,14^.

The roles of EMT or EMT-TFs in cancer progression are not limited to the regulation of cancer cell migration/invasion and dissemination; instead, these factors play multiple pivotal roles, including regulating tumor initiation, cancer stem cell (CSC) plasticity, cell fate specification, therapy resistance, and immune evasion^3,6^, although the underlying molecular mechanisms remain unclear. Of particular interest is the relationship between EMT, CSC status, and malignant progression. Certain types of cancer cells, such as breast, colorectal, ovarian, pancreatic, and prostate cancer cells, can acquire tumor-initiating capability after induction of EMT programs^6^. Overexpression of SNAI1, SNAI2, or TWIST in immortalized or transformed human mammary epithelial cells leads to a stem cell phenotype, which facilitates sphere formation and induces the expression of stem cell markers^1^. ZEB1 also confers stem cell-like properties and tumorigenicity on mouse and human pancreatic and colorectal cancer cells by suppressing stemness-inhibiting miRNAs such as miR-203, miR-200s, and miR-183, which repress the expression of stemness factors such as BMI1, SOX2, and KLF4^15^. TWIST1 upregulates BMI1, which is essential for promoting EMT and tumor-initiating capability in head and neck squamous cell carcinoma^16^ and hepatocellular carcinoma^17^ cells. Elevated SLUG expression correlates with the overexpression of stem-like genes, including CD133 and BMI1, in basal-type breast tumors^18^. SLUG is required for SOX9 stabilization, which supports CSCs and promotes metastasis in lung cancer^19^.

Malignant progression of most types of carcinoma depends on tumor cell EMT. In the context of metastasis, while EMT is involved in invasion, dissemination, and extravasation, the reverse process (mesenchymal-epithelial transition (MET)) is induced by either cell-intrinsic or stromal components; this process is thought to be required for metastatic colonization by disseminated cancer cells^1,3^. In this regard, it has been suggested^6,20^ that due to the multilayered regulatory network, EMT is a dynamic program that is (usually) partially activated and likely to generate cells residing in an intermediate, partially epithelial and partially mesenchymal phenotypic state (referred to as epithelial-mesenchymal plasticity (EMP)). This partial EMT phenotypic state is highly variable, changing based on the context, even within individual tumors. Partial/incomplete EMT or EMP is widely observed during development, wound healing, and cancer^6,21^. Importantly, cancer cells that undergo partial EMT are thought to have higher metastatic potential than fully mesenchymal cells, partially due to their ability to migrate in clusters and enhanced attachment to the ECM. Induction of the partial EMT phenotype is also associated with CSC expansion and subsequent metastatic colonization, as well as resistance to chemotherapy and targeted therapies^6,20^.

## The type II transmembrane serine protease (TTSP) family

Proteases are involved in almost all biological processes. The human genome contains approximately 700 peptidases (known and putative) in the MEROPS database^22^; among these, 178 are serine proteases, most of which (138) belong to the S1 family of trypsin-like proteases^23^. Serine proteases play critical roles in digestion, blood coagulation, fibrinolysis, development, immunity, tissue regeneration, and inflammation^23^. Dysregulation of protease activity causes cardiovascular and inflammatory diseases, cancer, osteoporosis, and neurological disorders^24,25^.

Most members of the serine protease family are either secreted or compartmentalized in cytoplasmic organelles. A subgroup of serine proteases is anchored directly to the plasma membrane. These cell surface-anchored serine proteases are tethered via a C-terminal transmembrane domain (type I), an amino-terminal transmembrane domain (type II), or a C-terminal hydrophobic region that functions as a signal for membrane attachment via a glycosyl-phosphatidylinositol linkage (GPI-anchored)^26^.

TTSPs, the largest group of membrane-anchored serine proteases, share a common structural architecture comprising a short intracellular N-terminal domain followed by a single-pass hydrophobic transmembrane domain, a variable-length stem region containing modular structural domains, and a C-terminal extracellular proteolytic domain that is displayed on the cell surface. The catalytic domain of TTSPs belongs to the S1 family of serine proteases^26^. Enteropeptidase (also known as enterokinase), which was identified over a century ago due to its critical role in food digestion, was the first identified TTSP (it was identified three decades ago by molecular cloning of enteropeptidase cDNA). TMPRSS2, TMPRSS11D/HAT, corin, and matriptase were subsequently identified as cell surface-anchored proteases^27^.

Humans express 17 TTSPs, which are divided into four different subfamilies: the (i) matriptase, (ii) hepsin/transmembrane protease, serine (TMPRSS), (iii) human airway trypsin-like (HAT)/differentially expressed in squamous cell carcinoma (DESC), and (iv) corin subfamilies^28^. The proteolytic domain is highly conserved between different TTSPs, and their proteolytic activity is dependent on a catalytic triad comprising His, Asp, and Ser. TTSPs possess a conserved Asp residue at the bottom of the S1 substrate-binding pocket, resulting in a cleavage preference for substrates that have Arg or Lys in the P1 position. TTSPs are expressed as single-chain, inactive zymogens that are activated proteolytically by cleavage of a positively charged residue (Arg or Lys) located in a conserved activation motif between the prodomain and catalytic domain; cleavage releases the active mature protein. After activation, they are predicted to remain bound to the cell membrane through conserved disulfide bonds that link the catalytic domain to the rest of the extracellular domain. Several TTSPs, including matriptase, hepsin/TMPRSS1, TMPRSS2, TMPRSS3, TMPRSS4, matriptase-2/TMPRSS6, and TMPRSS13, are capable of autoactivation^26,29^, indicating that their respective zymogens have baseline activity. On the other hand, the soluble forms of several TTSP members have been detected in vivo, implying that the extracellular domains may also be shed from the cell surface^30^. For example, in prostate cancer, soluble TMPRSS2 is generated by autocatalytic cleavage and released into the circulation^31^. In addition, TTSPs are regulated by endogenous protease inhibitors such as Kunitz domain-containing inhibitors and the serpin family. The membrane-associated Kunitz-type inhibitors hepatocyte growth factor activator inhibitor type I (HAI-1) and HAI-2 are thought to inhibit matriptase and hepsin^32^.

Expression of TTSPs is widespread throughout the human body and is enriched in epithelial tissues of various origins. Several members of the TTSP family play critical biological roles in diverse processes, including epithelial differentiation and barrier function, tissue homeostasis, iron metabolism, hearing, and blood pressure regulation^33^. Their dysregulated expression has been associated with diverse disorders, including cancer, skin defects, anemia, deafness, hypertension, and obesity^34^.

### TTSPs in cancer: matriptase

Several TTSPs are associated with cancer progression; indeed, early reports show that several members of the TTSP family are highly and selectively expressed by a variety of tumors relative to the corresponding normal tissues, indicating their potential as novel markers of tumor development and progression. In addition to expression analysis and basic biochemical characterization, studies have examined the functions of TTSPs in physiological and disease-related conditions, focusing on the identification of proteolytic substrates, deregulation of proteases, characterization of cognate inhibitors, and dissection of underlying intracellular signaling pathways. More recently, studies of genetically modified mice and mice treated with protease inhibitors have demonstrated that the dysregulated expression of TTSPs is causally associated with cancer initiation and progression^26,28,33,35^.

Matriptase, also known as MT-SP1, CAP3, TADG15, or epithin (gene name: ST14), is the most studied member of the TTSP family. Matriptase is expressed in the epithelial compartment in a wide variety of tissues and is upregulated in breast, prostate, ovarian, uterine, colon, cervical, gastric, head and neck, pancreatic, and skin cancers. Increased expression of matriptase is associated with a poor prognosis. In several cancers, the ratio of matriptase to its endogenous inhibitors HAI-1 and HAI-2 is increased, whereas the ratio is low in normal tissues; this suggests that an imbalance between matriptase and its endogenous inhibitors contributes to tumorigenesis^28,33^. Mice with transgenic expression of matriptase in the epidermis develop spontaneous squamous cell carcinoma and display susceptibility to carcinogen-induced tumorigenesis. In addition, matriptase-mediated tumorigenesis is completely blocked or impaired in double transgenic mice that concomitantly express transgenic epidermal HAI-1 or HAI-2, demonstrating the tumor-promoting role of matriptase/inhibitor imbalance^36^.

A study based on a mouse model of invasive ductal mammary carcinoma (MMTV-PymT) showed that matriptase hypomorphic mice with low levels of matriptase display a significant delay in tumor formation and blunted tumor growth due to reduced cancer cell proliferation^37^. Matriptase critically contributes to breast cancer progression via activation of the HGF/c-MET signaling pathway; upon proteolytic cleavage by matriptase, pro-HGF is converted to active HGF, which initiates c-MET signaling and activates downstream targets such as PI3K/AKT and Gab1, thereby contributing to cancer progression^37^. Among TTSPs, matriptase displays the most efficient pro-HGF processing activity^32^; it also activates pro-forms of urokinase plasminogen activator (uPA), macrophage stimulating protein 1 (MSP-1), protease-activated receptor 2 (PAR-2), and prostasin. Activation of uPA, MSP-1, and PAR-2 initiates downstream signaling pathways, such as plasmin activation, the MSP-1/RON pathway, and the PAR-2/NF-κB inflammatory pathway, respectively. In addition, other cancer-related proteins, including SRC-associated protein CUB domain-containing protein 1 (CDCP1/SIMA135/TRASK), platelet-derived growth factor (PDGF)-C and PDGF-D, vascular endothelial growth factor receptor 2 (VEGFR2), and insulin-like growth factor-binding protein-related protein-1 (IGFBP-rP1), are potential substrates of matriptase^38^. Active matriptase degrades the ECM components fibronectin and laminin in vitro^28^, suggesting a role in direct remodeling of the ECM and cancer cell invasion.

Matriptase is expressed widely in various organs and is critical for organ development. Matriptase-deficient mice are perinatal lethal and exhibit abnormal terminal differentiation of epidermal keratinocytes, increased thymocyte apoptosis, and loss of epidermal barrier function, leading to fatal postnatal dehydration^39^. These effects should be considered carefully if matriptase blockade is to be developed as an anticancer therapy.

### Role of TTSPs in viral infections

TTSPs expressed in the human respiratory tract are implicated in viral spread due to their ability to cleave the surface proteins of the virus, which mediates virus entry into cells via direct fusion at the plasma membrane surface. Thus, some TTSPs expressed in the human airway facilitate the entry and spread of influenza viruses and coronaviruses, including SARS-CoV-2. TMPRSS2, matriptase, TMPRSS11A, DESC1, TMPRSS11D/HAT, and TMPRSS13 cleave the spike protein of SARS-CoVs and/or MERS-CoVs in vitro, allowing the viruses to enter and replicate in airway epithelial cells^34,40^. TMPRSS2 is a potential target for the treatment of COVID-19 due to its involvement in viral spike protein processing and localization in human epithelial tissues of the respiratory, genitourinary, and gastrointestinal tracts and its coexpression with the coronavirus receptor hACE2 in lung tissues. TMPRSS2 mediates viral entry through two distinct mechanisms: proteolytic cleavage of ACE2, which enhances viral uptake, and cleavage of the spike protein, which triggers host cell entry via membrane fusion^41^. Mice lacking TMPRSS2 exhibit no abnormalities in the absence of infection^42^, suggesting that blocking TMPRSS2 may not have significant undesired side effects. Thus, inhibitors of TMPRSS2 may be a potential treatment for COVID-19. TTSPs with similar cleavage specificity are coexpressed in many tissues, including the human airway^34^, implying functional redundancy at certain levels. Therefore, TMPRSS2 may not be the sole protease that controls spike priming; hence, blocking TMPRSS2 alone may not be effective.

Similar to the coronavirus spike protein, the surface protein of influenza A and B viruses (hemagglutinin (HA)) also requires cleavage by host proteases to mediate entry into respiratory epithelial cells. Among TTSPs, TMPRSS2, TMPRSS4, matriptase, TMPRSS11A, TMPRSS11D/HAT, DESC1, and TMPRSS13 can cleave HA in vitro or in vivo^34,40,43^.

## Transmembrane protease serine 4 (TMPRSS4)

### Expression and regulation of TMPRSS4 in cancer

TMPRSS4 (gene ID 56649; chromosomal location 11q23.3), initially referred to as TMPRSS3 but also known as CAP2 or MT-SP2, was originally identified as a gene strongly expressed by most pancreatic carcinoma tissues but not in the normal pancreas or in cases of chronic pancreatitis^44^. Among the seven isoforms reported thus far, isoform 1 represents the longest transcript, encoding a 437 amino acid protein that contains an extracellular region harboring a trypsin-like serine protease domain. In humans, TMPRSS4 mRNA has been detected in the esophagus, stomach, small intestine, colon, bladder, and kidney^44^, although the physiological roles of TMPRSS4 remain unclear. Based on an analysis of The Cancer Genome Atlas (TCGA) and Genotype-Tissue Expression (GTEx) datasets, Katopodis et al.^45^ reported that TMPRSS4 is upregulated significantly in 11 types of cancer, including lung adenocarcinoma, lung squamous cell carcinoma, cervical squamous cell carcinoma, thyroid carcinoma, ovarian cancer, cancer of the rectum, pancreatic cancer, colon and stomach adenocarcinoma, uterine carcinosarcoma, and uterine corpus endometrial carcinoma, compared with normal control tissue but is downregulated in six types of cancer, including kidney carcinomas, acute myeloid leukemia, skin cutaneous melanoma, and testicular germ cell tumor. Furthermore, the authors used in silico tools to demonstrate specific expression within the central nervous system^45^. Elevated expression of TMPRSS4 correlates with poor prognosis of patients in various cancers (Table 1). Overexpression of TMPRSS4 is associated with stage progression and metastasis of multiple cancers, including colorectal^46–48^ and gastric cancers (Kim, unpublished results), suggesting a potential role for TMPRSS4 in the progression of noninvasive tumors to invasive malignancies.

**Table 1:** Expression of TMPRSS4 in human tumors.

Gene	Cancer type	Associated clinicopathological features	Ref.
*TMPRSS4*	NSCLC	Poor survival (OS)^a^ Poor survival (OS, RFS) Poor survival (OS, RFS)	^49,50,63^
	Breast cancer	Poor survival (OS, DFS), LN metastasis, histological grade^b^; Poor survival, tumor size, histological grade, LN metastasis, stage; Poor survival (OS, DFS), LN metastasis, grade, tumor size	^88–90^
	Gastric cancer	Poor survival, LN and distant metastases; Poor survival (OS, RFS); Poor survival, LN metastasis	^91–93^
	Gallbladder cancer	Poor survival (OS), LN metastasis, tumor size, histological grade	^94^
	Cervical cancer	Poor survival (OS, DFS), LN metastasis, stromal invasion, tumor grade	^95^
	Colorectal cancer	Poor survival (OS, DFS), LN and distant metastases; LN metastasis, stage; Poor survival (OS, DFS), metastasis, stage	^47,48,96^
	Prostate cancer	Poor survival (DFS)	^62^
	Hepatocellular carcinoma	Poor survival (OS), recurrence, stage	^9^
	Pancreatic cancer	Poor survival (OS)	^81^
	Thyroid cancer	LN metastasis, stage	^71^

^a^Squamous cell carcinoma.

^b^Triple-negative breast cancer. *OS* overall survival, *RFS* relapse-free survival, *DFS* disease-free survival, *LN* lymph node.

The factors underlying TMPRSS4 upregulation and the mechanism(s) by which TMPRSS4 expression is regulated remain unclear. According to Villalba et al.^49,50^, high expression of TMPRSS4 protein in non-small cell lung cancer (NSCLC) patients is significantly associated with reduced relapse-free survival and overall survival. Bioinformatic analyses using public databases (COSMIC, CCLE, IGDB.NSCLC) show that in NSCLC, genetic alterations (i.e., gene amplification, mutations, and/or rearrangements) in TMPRSS4 are infrequent. Instead, aberrant hypomethylation in tumors correlates with high TMPRSS4 expression and is an independent prognostic predictor. Treatment of TMPRSS4-negative cells with a demethylating agent induces the expression of TMPRSS4^49^. Although the TMPRSS4 promoter does not contain canonical CpG islands, it does contain relevant methylation regions, including CpGs located at positions −116 bp to +271 bp relative to the transcription start site (TSS). Loss of TMPRSS4 promoter methylation from CpGs spanning −116 bp to +271 bp relative to the TSS predicts a poor outcome for NSCLC patients. Analysis of transcription factor-binding site prediction databases suggests possible involvement of transcription factors related to proliferation, EMT, and inflammation; for example, binding of ZEB1, E2F1, Myc, NF-κB, and STAT3 close to these CpGs can be mediated by hypomethylation^49^, although further experimental studies are needed to determine how TMPRSS4 expression is modulated.

Furthermore, Villalba et al.^50^ showed that the expression of TMPRSS4 protein is an independent prognostic factor for NSCLC, particularly for patients with stage I cancer. The authors obtained plasma and bronchoalveolar lavage samples from healthy individuals and patients and measured the copy number of methylated and unmethylated CpGs within the TMPRSS4 promoter. They found that TMPRSS4 methylation status can be used as a diagnostic tool for early-stage patients and to monitor relapse in surgically resected patients. These results suggest that TMPRSS4 is a potential biomarker that can be used to identify patients at high risk of recurrence^50^. In addition, the hypomethylation status of TMPRSS4 could be used as a companion diagnostic marker for TMPRSS4-targeted therapy in the future.

In general, epigenetic changes occur at an early stage of tumor development. Compared with that in normal tissue, the expression of TMPRSS4 mRNA in atypical adenomatous hyperplasia, a known precursor for lung adenocarcinoma, is moderately (by 2-fold) upregulated, and it is further (by 8-fold) upregulated in early-stage lung adenocarcinoma, according to the data published by Sivakumar et al.^51^. Therefore, it is likely that epigenetic regulation may contribute to the upregulation of TMPRSS4 expression and that TMPRSS4 upregulation occurs at the premalignant and early stages of NSCLC. In contrast, overexpression of TMPRSS4 is associated with stage progression and metastasis of colorectal and gastric cancers. Consistent with this, analysis of TCGA datasets revealed that TMPRSS4 DNA is hypomethylated in certain cancer types, including NSCLC and pancreatic cancer, but not in colorectal or gastric cancers (Kim, unpublished results).

Expression of TMPRSS4 by NSCLC cells increases under hypoxic conditions^52^, suggesting that the hypoxic tumor microenvironment promotes TMPRSS4 expression. Consistent with this, a tripeptide, tyroserleutide, inhibits the irradiation-induced invasiveness and metastatic potential of HCC cells by downregulating HIF-1α and TMPRSS4^53^. TMPRSS4 expression in NSCLC samples correlates negatively with that of tissue factor pathway inhibitor 2 (TFPI-2), and TFPI-2 partially inhibits transcription of TMPRSS4, leading to reduced lung cancer cell growth^54^. However, further investigations are needed. Several reports have shown that miRNAs or lncRNAs regulate TMPRSS4. For example, lncRNA HOXA11-AS acts as a tumor promoter in breast cancer by regulating the miR-125a-5p/TMPRSS4 axis^55^. miR-125a-p5, a key regulator in carcinogenesis that is expressed at abnormal levels by specific types of cancer, including NSCLC, colorectal, pancreatic, and prostate cancers, can target the 3′-UTR of TMPRSS4 mRNA to downregulate TMPRSS4 expression directly, resulting in reduced growth and enhanced apoptosis of lung cancer cells^56^. miR-1258, which plays an anticancer role in various cancers, can target TMPRSS4 directly and is associated with malignant progression of papillary thyroid carcinoma^57^. miR-541-3p can directly target and inhibit TMPRSS4 expression in HCC, thereby suppressing the invasion and migration of HCC cells^58^. miR-551b-3p, the expression of which is inhibited by lncRNA SMARCC2, functions as a tumor suppressor by directly suppressing TMPRSS4 expression, as well as the proliferation, motility and invasiveness of gastric cancer cells^59^.

### Functions of TMPRSS4 during the regulation of EMT and CSCs

While the physiological function and in vivo substrates of TMPRSS4 remain unidentified, much of our knowledge about TMPRSS4 originates from clinical analysis and cell- and xenograft-based studies showing a correlation with cancer and other diseases. Indeed, a mutation in TMPRSS4 is associated with autosomal recessive cerebral atrophy syndrome, a novel pediatric neurodegenerative disorder^60^.

Knockdown of TMPRSS4 in lung, colon, and prostate cancer cells reduces cell migration and invasion through the ECM and suppresses proliferation, while overexpression increases cell migration and invasion of lung, colon, and prostate cancer cells and proliferation and anchorage-independent growth of lung and prostate cancer cells^8,61,62^. Reduction of TMPRSS4 expression in lung and prostate cancer cells inhibits tumor growth and metastasis in xenograft nude mouse models^63,64^, while overexpression of TMPRSS4 in lung, prostate, and colon cancer cells promotes tumor growth and metastasis in vivo^62,64,65^. In colon, prostate, and lung cancer cells, TMPRSS4 promotes the downregulation of E-cadherin, which is often accompanied by morphological changes and actin rearrangement, leading to EMT events and cancer cell invasion. TMPRSS4 also modulates cell-matrix adhesion and cell spreading by modulating integrins such as α5β1 and α4β1, which are implicated in EMT, cell motility, and/or cell survival^8,64^.

Among intracellular signaling mediators, TMPRSS4 activates the focal adhesion kinase (FAK)/c-Src, AKT, and ERK signaling pathways, mainly through upregulation of and possible interaction with integrin α5, leading to EMT and invasiveness. Consistent with this, expression of TMPRSS4 in human colorectal cancer tissues correlates positively with enhanced expression of integrin α5 and negatively with expression of E-cadherin, thereby confirming TMPRSS4-mediated regulation of EMT^46^. Larzabal et al.^66^ reported that miR-205 is involved in TMPRSS4-mediated upregulation of integrin α5 expression and metastasis of NSCLC cells, thereby supporting a link between TMPRSS4 and integrin α5. Cheng et al.^67^ reported that HAI-1 suppresses EMT in pancreatic cancer cells by modulating both matriptase and TMPRSS4, suggesting that TMPRSS4 activity may be regulated by the endogenous inhibitor HAI-1, although further investigation is needed to determine whether HAI-1 interacts with/inhibits TMPRSS4 directly. In addition, xenograft model studies have shown that TMPRSS4-mediated EMT plays a critical role in radiation-induced long-term metastasis of residual HCC^68^.

TMPRSS4 activates JNK and ERK1/2, leading to activation of the transcription factors activator protein-1 (AP-1) and SP1/3^61^. TMPRSS4 also activates NF-κB in lung, prostate, and colon cancer cells, possibly through FAK- or AXL-mediated activation of AKT^64,69^. Consistent with this, TMPRSS4 promotes the invasion of gastric cancer cells^70^ and the growth of lung cancer cells^56^ by activating NF-κB signaling, although the precise mechanisms need to be determined. In addition, TMPRSS4 may be involved in regulating the tumor microenvironment (e.g., regulating the immune status or angiogenesis) via NF-κB, thereby contributing to malignancy; again, these possibilities require further investigation.

TMPRSS4 upregulates EMT-TFs such as ZEB2 (at the mRNA level), SLUG, and TWIST1 in a context/cell-dependent manner via MAPK-mediated activation of AP-1 and SP1^64^. TMPRSS4 upregulates SLUG and cyclin D1 in prostate cancer cells by activating AP-1, leading to both invasion and proliferation. In addition, a positive feedback loop between SLUG and AP-1 promotes the expression of cyclin D1 and increases cell proliferation. SLUG activates AP-1 by upregulating AXL expression and signaling^62^. Consistent with this, TMPRSS4 promotes thyroid cancer cell proliferation via CREB phosphorylation and transcription of cyclin D1^71^.

TMPRSS4 confers stem-like properties on prostate and colon cancer cells, inducing aldehyde dehydrogenase (ALDH) activation, tumorsphere formation, and resistance to chemotherapeutics and anoikis, thereby increasing the survival of circulating tumor cells and promoting early metastasis^64^. These features are accompanied by upregulation of the stemness-related factors SOX2, BMI1, CD133, and the ABC transporter MDR1/ABCB1. SOX2, a pluripotency-inducing transcription factor, is overexpressed in almost all human cancer types and is associated with a poor prognosis; it is also considered to be a key regulator of tumorigenicity, drug resistance, and metastasis^72^. SLUG and TWIST1 contribute to TMPRSS4-mediated CSC-like features through upregulation of SOX2. TWIST1 upregulates the transcription of SOX2 by interacting with the proximal E-box element in the SOX2 promoter, while SLUG binds to and stabilizes SOX2 to prevent proteasomal degradation. Clinically, expression of TMPRSS4 correlates with levels of ALDH, SOX2, PROM1, SNAI2, and TWIST1. Analysis of CCLE data reveals that SOX2 expression correlates positively with that of TWIST1 but not that of other EMT-inducing transcription factors^64^. TMPRSS4 contributes to tumor growth and metastatic seeding through diverse molecular mechanisms, thereby linking EMT and tumorigenic programs.

Similarly, De Aberasturi et al.^73^ reported that increased expression of CSC markers such as ALDH and OCT-4 correlates positively with expression of TMPRSS4 in NSCLC. In addition, overexpression of TMPRSS4 confers EMT features and CSC-like properties on lung cancer cells, accompanied by enhanced tumorigenicity in vivo. Overexpression of TMPRSS4 also causes cancer cells to become more resistant to chemotherapeutics^73^, while downregulation of TMPRSS4 significantly increases sensitivity to chemotherapeutics by impairing proliferation^65^. In addition, Villalba et al.^74^ recently performed large-scale analyses of five public NSCLC datasets and identified a synthetic lethal interaction between TMPRSS4 and DDR1; TMPRSS4/DDR double knockdown resulted in cell cycle arrest and apoptosis; furthermore, cells became highly sensitized to cisplatin in vitro, and tumors regressed in vivo.

Therefore, TMPRSS4 expression in a variety of different cancers is associated with poor prognosis and survival, which may be due to an increase in the CSC population. Further studies should investigate whether TMPRSS4 promotes tumor initiation in transgenic animal models.

### Precursor substrates and signals regulated by TMPRSS4

Many studies have investigated the substrates of TTSPs. During tumor progression, TTSPs such as matriptase, hepsin, and TMPRSS2 activate pro-HGF to elicit the HGF/c-MET pro-oncogenic/cell survival signaling pathway in vivo and/or in vitro^28,33^. Matriptase and hepsin cleave and activate pro-MSP-1 and pro-uPA. Prostasin, a GPI-anchored protein, is also a substrate of matriptase and hepsin. The PAR-2/NF-κB inflammatory pathway is activated by matriptase, hepsin, and TMPRSS2^28^.

When coexpressed with the mouse epithelial sodium channel (ENaC) in the *Xenopus* oocyte system, TMPRSS4, matriptase, and prostasin activate ENaC through proteolytic cleavage and subsequent removal of an inhibitory moiety from its γ-subunit, thereby regulating sodium and water flux across the high-resistance epithelium^39,75^. In zebrafish embryos, TMPRSS4 is necessary for organogenesis; indeed, knockdown of TMPRSS4 using morpholinos results in severe defects in tissue development and cell differentiation, including disturbed skeletal muscle formation, a decelerated heartbeat, and a degenerated vascular system^76^, suggesting that TMPRSS4 may modulate the activity of adhesion molecules involved in organ development. In contrast, TMPRSS4 knockout mice are viable and fertile and have no obvious abnormalities, suggesting functional redundancy of TMPRSS4 with respect to development and ENaC activation^77^. These data also suggest that targeted ablation of TMPRSS4 in cancer cells may have minimal side effects.

Similar to other TTSP family members, TMPRSS4 shows a preference for basic Lys and Arg residues at the P1 position, as shown by studies employing ENaC mutagenesis^78^ and focused peptide substrates^8^ (Kim, unpublished results). TMPRSS4 cleaves the γ-subunit of ENaC within a region of seven basic residues, thereby producing a unique ~70 kDa carboxyl-terminal fragment of the γ-subunit^78^. Regarding the precursor substrates cleaved by TMPRSS4 during tumor progression, Min et al.^79^ demonstrated that TMPRSS4 converts inactive pro-uPA into the active form directly through its serine proteolytic activity to promote cancer cell invasion. Consistent with this, TMPRSS4 promotes cancer cell invasion in a manner that is dependent on serine proteolytic activity^79^, and small-molecule compounds inhibiting TMPRSS4 serine protease activity reduce TMPRSS4-dependent invasion^69^. Furthermore, TMPRSS4 upregulates transcription of the uPA gene in prostate and lung cancer cells by activating AP-1 and SP1/3 in a MAPK (mainly JNK)-dependent manner. Expression of TMPRSS4 shows a strong positive correlation with uPA expression in human lung and prostate adenocarcinoma. In addition, TMPRSS4 interacts with the uPA receptor (uPAR/CD87) to activate the JNK, ERK, and c-Src signaling pathways in prostate and lung cancer cells^61^.

uPA is a well-known serine protease involved in invasion and metastasis; it catalyzes the conversion of inactive plasminogen into active plasmin, which can degrade most ECM components and activate MMPs to promote invasion. Increased levels of uPA correlate with a poor prognosis in many types of cancer, including breast, lung, stomach, bladder, colon, prostate, and ovarian cancers. Complexes of uPA and uPAR on the tumor cell surface interact with coreceptors (e.g., integrins) to activate intracellular signaling pathways, including MAPK, PI3K/AKT, Rac1, and JAK/STAT, which promote cell migration, invasion, survival, metastasis, EMT, stem cell-like properties, and chemotherapy resistance^80^. Both uPA and uPAR are involved in TMPRSS4-mediated invasion. TMPRSS4 activates the JNK, ERK, and c-Src signaling pathways, possibly through its interaction with uPAR, leading to activation of AP-1 and SP1 and subsequent expression of uPA. These results suggest that TMPRSS4 acts as an upstream regulator of the uPA/uPAR system by upregulating pro-uPA at both the transcriptional and posttranslational levels. The association between TMPRSS4 and uPAR and the subsequent modulation of cell signaling may be a novel mechanism controlling EMT, invasion, CSCs, and drug resistance. In the future, substrates of TMPRSS4 other than pro-uPA should be investigated both in vivo and in vitro. In addition, it would be worth investigating whether TMPRSS4 has a pro-oncogenic function and activates signaling pathways in a protease activity-independent manner.

Recently, Gu et al.^81^ showed that TMPRSS4 promotes cell proliferation and inhibits apoptosis in pancreatic ductal adenocarcinoma by activating the ERK1/2 signaling pathway, suggesting a pro-oncogenic role for TMPRSS4. Dong et al.^82^ showed that TMPRSS4 increases the expression of the heparin-binding-EGF precursor and promotes its proteolytic cleavage by enhancing MMP-9 expression through EGFR/AKT/mTOR/HIF-1α signaling, leading to angiogenesis, proliferation, and invasion in HCC.

### Role of TMPRSS4 in viral infections and lung fibrosis

According to GTEx RNA-seq data, three TTSPs (TMPRSS2, matriptase, and hepsin) are expressed at high levels in healthy human lungs, whereas TMPRSS3 and TMPRSS13 are expressed at lower levels, and other TTSPs, including TMPRSS4, are expressed at very low levels or not at all^34^. At present, the levels of TMPRSS4 protein in normal tissues, including lung, remain to be determined. However, Zang et al.^83^ showed that TMPRSS2, matriptase, and TMPRSS4 are highly expressed in human intestinal epithelial cells, and TMPRSS4 and TMPRSS2 (but not matriptase) promote entry of SARS-CoV-2 into human small intestinal enterocytes. TMPRSS4 cleaves the spike protein, although less efficiently than TMPRSS2, in vitro^83^. In addition, TMPRSS2 and TMPRSS4 play a role in the spread of influenza virus by cleaving and activating HA. Transient expression of TMPRSS2 and TMPRSS4 in host cells facilitates cleavage of HA and replication of influenza virus in vitro^43^. Consistent with this, TMPRSS2 and TMPRSS4 double-knockout mice show a marked reduction in the spread of H3N2 influenza virus, whereas deletion of TMPRSS4 or TMPRSS2 alone does not or only slightly protects them from death upon infection with H3N2 influenza virus, although TMPRSS2-deficient mice are protected against H1N1 influenza virus infection^84^. Additionally, TMPRSS4 is strongly upregulated in the lungs of mice infected with H1N1 influenza virus^85^.

Among all TTSPs, matriptase and TMPRSS4 may play profibrotic roles in the lung that could contribute to the pathogenesis of idiopathic pulmonary fibrosis (IPF)^34^. TMPRSS4 is upregulated in the lungs of IPF patients and in a bleomycin-induced pulmonary fibrosis mouse model. TMPRSS4 is upregulated and expressed mainly by mast cells and alveolar epithelial cells, primarily in fibrotic areas of IPF lungs. Furthermore, TMPRSS4 deficiency attenuates bleomycin-induced lung fibrosis in mice^86^. However, the mechanisms underlying the profibrotic activity of TMPRSS4 are unclear.

### Development of TMPRSS4 inhibitors

Modulating TMPRSS4 activity may be a treatment for several types of cancer. Screening of a small-molecule compound library identified 2-hydroxydiarylamide derivatives that inhibit TMPRSS4 serine protease activity in biochemical assays using a recombinant TMPRSS4 serine protease and a fluorogenic substrate^87^. Among these derivatives, IMD-0354, a selective IκB kinase (IKK)-β inhibitor that is effective in acute and subacute inflammatory disease, displayed an IC_50_ of 11 µM, and KRT1853, a novel derivative of IMD-0354, displayed twofold stronger inhibition^87^. IMD-0354 and KRT1853 inhibited the effects of TMPRSS4-mediated cellular signaling, including activation of SP1, AP-1, and NF-κB and induction of bcl-2 and survivin. Both compounds efficiently reduced cancer cell invasion and proliferation and induced apoptosis, although the possibility that the compounds modulate the activity of other serine proteases cannot be ruled out. Importantly, KRT1853 efficiently suppressed tumor growth in nude mice bearing prostate and colon cancer xenografts^69^. At present, the crystal structure of TMPRSS4 is not available. When it becomes available, it would be worth investigating the structure of the TMPRSS4 inhibitor-docking protease domain. In terms of drug repurposing, KRT1853 and IMD-0354 may be useful anticancer agents or good bases for further development.

## Concluding remarks

**Fig. 1:**
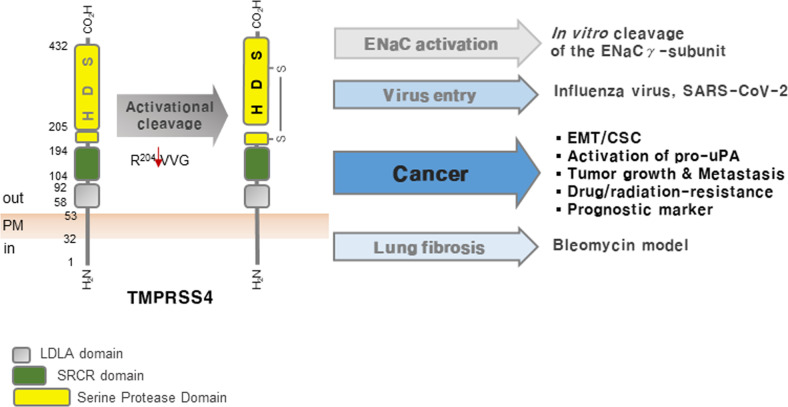
Biological roles of TMPRSS4 in health and disease. After being synthesized and translocated to the plasma membrane, TMPRSS4 is autoactivated by cleavage between R204 and V205. In the *Xenopus* oocyte system, TMPRSS4 activates ENaC by removing an inhibitory peptide of the γ-subunit. However, TMPRSS4-deficient mice show no abnormalities, suggesting functional redundancy in development, homeostasis, and ENaC activation. Under pathological conditions, TMPRSS4 cleaves the HA molecule of influenza virus to promote viral spread in the lung; it can also cleave the spike protein of SARS-CoV-2 to promote viral entry into intestinal enterocytes. Clinically, TMPRSS4 is a prognostic marker for diverse types of cancer and exhibits protumorigenic and prometastatic activity. The underlying mechanisms include acquisition of EMT and CSC-like properties, which confer chemotherapy resistance. TMPRSS4 cleaves pro-uPA directly to yield active uPA, thereby acting as an upstream regulator of the uPA/uPAR system. TMPRSS4 is upregulated in IPF lungs and in a bleomycin-induced pulmonary fibrosis mouse model. The fibrotic response in TMPRSS4-deficient mice is attenuated, suggesting that TMPRSS4 may play a profibrotic role. However, the underlying mechanisms remain unknown. LDLA: low-density lipoprotein receptor class A domain; SRCR: scavenger receptor cysteine-rich domain.

Figure 1 summarizes the biological roles of TMPRSS4 in physiological and pathological contexts. Based on the expression patterns, biological roles, and structural characteristics of TMPRSS4, this druggable protease could be a novel therapeutic target in solid tumors. However, more studies of the functions and mechanisms are needed. In addition, genetically engineered mouse models are needed to fully unravel the functions of the signaling pathways summarized in this review. The TMPRSS4 knockout mice generated by Keppner et al.^77^ will be useful for future cancer studies. Due to the high similarity of the active sites among different serine proteases, it will be a challenge to develop a highly selective inhibitor of TMPRSS4 targeting the active site.
